# Carbon dioxide dynamics in relation to neurological outcome in resuscitated out-of-hospital cardiac arrest patients: an exploratory Target Temperature Management Trial substudy

**DOI:** 10.1186/s13054-018-2119-5

**Published:** 2018-08-18

**Authors:** Florian Ebner, Matt B. A. Harmon, Anders Aneman, Tobias Cronberg, Hans Friberg, Christian Hassager, Nicole Juffermans, Jesper Kjærgaard, Michael Kuiper, Niklas Mattsson, Paolo Pelosi, Susann Ullén, Johan Undén, Matt P. Wise, Niklas Nielsen

**Affiliations:** 1 Department of Anesthesia and Intensive Care, Helsingborg Hospital, Helsingborg, Sweden; 2 Department of Intensive Care, Liverpool Hospital, Locked Bag 7103, Liverpool BC, Sydney, NSW 1871 Australia; 3 Department of Intensive Care Medicine, Laboratory of Experimental Intensive Care and Anesthesiology, Academic Medical Center, University of Amsterdam, Meibergdreef 9, Amsterdam, 1105 AZ The Netherlands; 4 Department of Surgical Sciences and Integrated Diagnostics, Anesthesia and Intensive Care, San Martino Policlinico Hospital, University of Genoa, Genoa, Italy; 5 Intensive Care Unit, Leeuwarden Medical Centrum, Borniastraat 38, NL8934 AD Leeuwarden, The Netherlands; 6 Department of Anaesthesia and Intensive Care, Hallands Hospital, Halmstad, Sweden; 7 Department of Clinical Sciences, Neurology, Skåne University Hospital, Getingevägen 5, 221 85 Lund, Sweden; 8 Department of Anaesthesia and Intensive Care, Skåne University Hospital, Getingevägen 5, 221 85 Lund, Sweden; 9 Adult Critical Care, University Hospital of Wales, Heath Park, Cardiff, CF144XW UK; 10 Clinical Studies Sweden, Skåne University Hospital, Remissgatan 4, 221 85 Lund, Sweden; 11 Department of Cardiology, Rigshospitalet, University of Copenhagen, Copenhagen, Denmark; 12 Department of Clinical Medicine, University of Copenhagen, Copenhagen, Denmark

**Keywords:** Out-of-hospital cardiac arrest, Carbon dioxide partial pressure, Cerebral performance, Biomarker, Serum Tau

## Abstract

**Background:**

Dyscarbia is common in out-of-hospital cardiac arrest (OHCA) patients and its association to neurological outcome is undetermined.

**Methods:**

This is an exploratory post-hoc substudy of the Target Temperature Management (TTM) trial, including resuscitated OHCA patients, investigating the association between serial measurements of arterial partial carbon dioxide pressure (PaCO_2_) and neurological outcome at 6 months, defined by the Cerebral Performance Category (CPC) scale, dichotomized to good outcome (CPC 1 and 2) and poor outcome (CPC 3–5). The effects of hypercapnia and hypocapnia, and the time-weighted mean PaCO_2_ and absolute PaCO_2_ difference were analyzed. Additionally, the association between mild hypercapnia (6.0–7.30 kPa) and neurological outcome, its interaction with target temperature (33 °C and 36 °C), and the association between PaCO_2_ and peak serum-Tau were evaluated.

**Results:**

Of the 939 patients in the TTM trial, 869 were eligible for analysis. Ninety-six percent of patients were exposed to hypocapnia or hypercapnia. None of the analyses indicated a statistical significant association between PaCO_2_ and neurological outcome (*P* = 0.13–0.96). Mild hypercapnia was not associated with neurological outcome (*P* = 0.78) and there was no statistically significant interaction with target temperature (*P*_interaction_ = 0.95). There was no association between PaCO_2_ and peak serum-Tau levels 48 or 72 h after return of spontaneous circulation (ROSC).

**Conclusions:**

Dyscarbia is common after ROSC. No statistically significant association between PaCO_2_ in the post-cardiac arrest phase and neurological outcome at 6 months after cardiac arrest was detected. There was no significant interaction between mild hypercapnia and temperature in relation to neurological outcome.

**Electronic supplementary material:**

The online version of this article (10.1186/s13054-018-2119-5) contains supplementary material, which is available to authorized users.

## Background

Out-of-hospital cardiac arrest (OHCA) is a common reason for critical care admission [1–3]. Despite increasing survival after OHCA with initial shockable rhythm in the last decades [4, 5], overall survival is still low [6, 7], and brain injury continues to be the principal cause of death and disability [5, 8]. Guidelines recommend ventilation to normal carbon dioxide levels after return of spontaneous circulation (ROSC) [9, 10]. However, unintended hyperventilation during and after resuscitation frequently occurs and variability in ventilation leading to dyscarbia is a prevalent finding in the post-cardiac arrest phase [11, 12]. Elevated arterial carbon dioxide partial pressure (PaCO_2_), hypercapnia, may lead to cerebral vasodilatation and increased cerebral blood flow (CBF), while a decrease in PaCO_2_, hypocapnia, can exert the opposite effect [13]. PaCO_2_ is also a central variable in acid–base homeostasis encompassing hypercapnic acidosis or hypocapnic alkalosis [14]. In recent studies, PaCO_2_ was associated with neurological outcome after OHCA; hypocapnia was related to poor neurological outcome [15–17], whilst hypercapnia was associated with good as well as poor neurological outcome [16–21].

As hypercapnia has been associated with good neurological outcome [16], the novel concept of targeted therapeutic mild hypercapnia (TTMH) has been tested in the pilot randomized Carbon Control and Cardiac Arrest (CCC) trial and shown to be safe, feasible to perform, to lower biomarkers of brain damage, and to have a tendency for improved global functional outcome [21]. A phase III randomized clinical trial has recently started recruiting patients to further investigate the impact of TTMH on neurological outcome (ClinicalTrials.gov NCT03114033) and there are active plans to coenroll patients with the ongoing TTM2 trial (ClinicalTrials.gov NCT02908308) comparing hypothermia to normothermia after cardiac arrest [22].

We conducted this exploratory substudy of the TTM trial to describe the evolution of PaCO_2_ in serial measurements at predefined time points during the first hours after ROSC, to explore the role that PaCO_2_ may have in the neurological outcome of OHCA patients, and to specifically analyze the interaction between mild hypercapnia and targeted temperature management in relation to neurological outcome. To further strengthen the analyses, we investigated the association of PaCO_2_ to a surrogate marker of neurological outcome: peak levels of serum-protein Tau, a marker of neuronal damage that has shown to be more accurate than neuron specific enolase (NSE) in predicting outcome [23].

## Methods

This study is based on the TTM trial conducted between 2010 and 2013 and was approved by the TTM trial steering group before completion of the trial [24]. Ethical committees in each participating country approved the TTM trial protocol and informed consent was waived or obtained according to national legislations, in line with the Helsinki declaration. The patients included were unconscious (GCS < 8) adults (≥ 18 years of age) with stable ROSC after OHCA of presumed cardiac cause. The main exclusion criteria were unwitnessed cardiac arrest with asystole as the primary rhythm, known or suspected intracranial hemorrhage or stroke, and time from ROSC to screening > 240 min [24]. All patients were admitted to an intensive care unit (ICU), intubated, sedated, and mechanically ventilated. After inclusion, the patients were randomized to the 33 °C group or the 36 °C group and the temperature controlled during the intervention period of 36 h, which commenced at inclusion into the trial. Mandatory sedation was discontinued 36 h after inclusion. The TTM study analyzed 939 eligible patients with no difference in survival or neurological outcome at 6 months between the two allocation groups [24]. For this substudy we included patients surviving the intervention period in order to have a defined exposure period for carbon dioxide.

### Patient data and blood sampling

Prehospital data were reported according to the Utstein criteria [25]. Baseline, intervention-related, and physiological variables, comorbidities, demographic, prehospital, and admission data, as well as characteristics of the cardiac arrest and baseline laboratory analyses were collected. A complete arterial blood gas analysis was performed in all patients at admission to the hospital, at the start of the intervention (T0, which also was the time of randomization), and after 4, 12, 20, 28, 32, and 36 h. All arterial blood gases were managed according to the alpha-stat method. The median time from ROSC to randomization was 133 (interquartile range 83–188) min. To include the admission blood gas (after ROSC, but before randomization) we timed this PaCO_2_ value to 1 h before T0 (T – 1). PaCO_2_ data were surveyed for physiological plausibility and in four of the measurements we corrected a misplaced decimal point. Corrections were conducted by FE and NN in accordance with other data registered on the same patient.

### Outcome

The primary outcome was overall neurological function at 6-month follow-up, using the Cerebral Performance Category (CPC) scale (CPC 1 = good cerebral performance; CPC 2 = moderate cerebral disability, independent in activities of daily life; CPC 3 = severe cerebral disability, dependent on others for daily support; CPC 4 = vegetative state; and CPC 5 = dead) [26–28]. The CPC scale was dichotomized to good (CPC 1 and 2) and poor (CPC 3–5) outcome [29]. In a secondary analysis we used the biomarker protein Tau as the outcome to strengthen the analyses using neurologic functional outcome.

### Main analysis

#### Levels of carbon dioxide

As our main analysis, we studied the association of dyscarbia with neurological outcome by dividing the cohort into three groups according to the single highest or lowest PaCO_2_ value during the observation period. The groups were defined as hypocapnia (< 4.5 kPa), normocapnia (4.5–6.0 kPa), and hypercapnia (> 6.0 kPa) in keeping with a previous investigation [16]. The outcomes of the hypercapnia and hypocapnia groups were each compared with the normocapnia group. Then, we compared the outcome of the hypercapnia and hypocapnia groups with the outcome of a compound group of the remaining patients.

### Secondary analyses

#### Carbon dioxide amplitude

The amplitude in PaCO_2_ (∆PaCO_2_) was calculated as a continuous variable, investigating an association of maximum amplitude in PaCO_2_ during the observation period and neurological outcome.

#### Carbon dioxide over time

We obtained an approximation of the time-weighted mean carbon dioxide exposure as an area under the curve (AUC) by integrating PaCO_2_ over time. The AUC including eight PaCO_2_ values was analyzed, investigating an association with neurological outcome over the whole observation period, as well as the AUC of the first four PaCO_2_ values, in order to study the influence of early dyscarbia. To investigate nonlinear patterns, we divided the PaCO_2_ AUC for the whole exposure time into quintiles.

#### Maximum PaCO_2_ and lowest pH analysis

The association between maximum PaCO_2_ and lowest pH as continuous variables and neurological outcome was evaluated in univariable analyses. Thereafter, both variables were introduced into a combined logistic regression model.

#### Therapeutic targeted mild hypercapnia

From the time-weighted mean carbon dioxide exposure analysis, we extracted two PaCO_2_ groups approximating the PaCO_2_ ranges employed by Eastwood et al. [21] (4.5–6.0 kPa and 6.0–7.30 kPa) and subdivided them according to target temperature (33 °C and 36 °C) in order to detect possible interactions between PaCO_2_ and temperature in relation to outcome.

### Analyses using a biomarker as outcome

#### Association of PaCO_2_ and s-Tau

A nested cohort analysis was performed in 689 patients in a previous substudy of the TTM trial, evaluating s-Tau levels at 24, 48, and 72 h after ROSC, showing the highest accuracy of predicting poor outcome after 6 months for peak s-Tau at 48 and 72 h [23]. Therefore, we analyzed the association of PaCO_2_ and peak s-Tau at these time points, employing the multivariable models used for our primary analyses.

### Sensitivity analyses

For sensitivity analysis we used the complete case cohort consisting of 485 patients (56%) with blood gas samples registered from all measuring points and the total case (*n* = 939) cohort including 100% of the patients, even those not surviving the full exposure period.

### Statistical considerations

Proportions are expressed as percentages and continuous data as means with standard deviations (SDs). The association between PaCO_2_ and neurological outcome was analyzed using logistic regression. Except for the maximum PaCO_2_ and lowest pH analyses, all analyses were corrected for prespecified and relevant covariates: age (years), sex (male/female), chronic heart failure (yes/no), asthma/chronic obstructive pulmonary disease (yes/no), cardiac arrest witnessed (yes/no), bystander CPR (yes/no), first rhythm shockable (yes/no), time to ROSC (minutes), GCS-Motor score (1 versus 2–5), shock on admission (yes/no), and pH at admission (units). Whether pooling of the two temperature groups was feasible was established by a term of interaction model between the PaCO_2_ analysis groups and the two temperature groups. A significant term of interaction entailed subgroup analyses for the 33 °C and the 36 °C groups. A nonsignificant term of interaction entailed a combined group analysis.

Logistic regressions are presented as odds ratios (ORs) with 95% confidence intervals (CIs), with OR below 1 indicating better and OR above 1 indicating worse neurological outcome. ORs relating to continuous data describe the variation per one unit (1 kPa for PaCO_2_ and 1 unit for pH).

For s-Tau analysis, multivariable linear regression was used including the same covariables and interaction analyses as already described. s-Tau values where transformed to a logarithmic scale and used as outcome in the linear regression analyses. The regression coefficients achieved for each independent variable were transformed back and reflect the multiplicative change in s-Tau. This means that coefficients below 1 correspond to a decrease in s-Tau and coefficients above 1 to an increase. Linear regressions are presented as coefficient estimates with 95% CI.

Multiple imputation was used to compensate for missing values. We employed predictive mean matching, where information from nonmissing values of the same individual, all available TTM trial study variables, and values of matching patients were used to impute the missing values. In total, 20 imputed datasets were generated by chained equations and evaluated by graphical methods. The summary measures of PaCO_2_ (e.g., hypocapnia, hypercapnia, range) where computed for each imputed dataset and evaluated using regression models. The estimates from the logistic and linear regression for each imputed sample were combined into one estimate with 95% CI including the uncertainty from the multiple imputations based on Rubin’s rule [30]. The primary analyses were performed on a multiple imputation cohort. A complete case cohort was used for sensitivity analysis. We regarded two-sided *P* < 0.05 as significant. Analyses were conducted using IBM SPSS statistics for Windows version 22.0 (Armonk, NY, USA) and R: A language and environment for statistical Computing version 3.3.3 (R Foundation for Statistical Computing, Vienna, Austria), with the package *mice* used for multiple imputations [31].

## Results

**Fig. 1:**
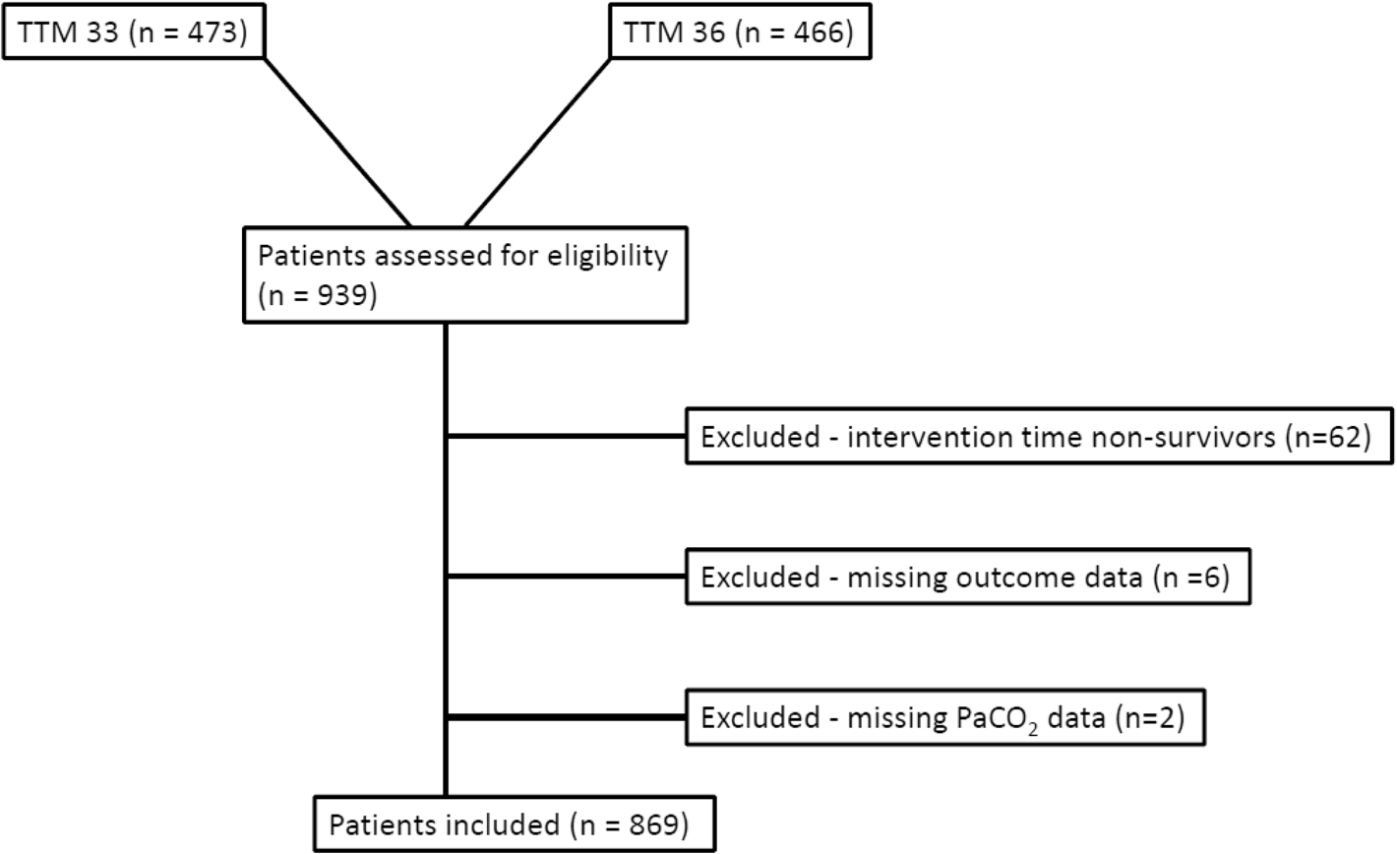
Patient selection pathway for the PaCO_2_ outcome analyses. TTM 33/TTM 36 TTM group at 33 °C/36 °C core body temperature derived from TTM trial study [24]. Selection pathway for s-Tau analysis or sensitivity analyses not shown. TTM target temperature management, *n* number of patients, PaCO_2_ partial carbon dioxide pressure

From the 939 patients included in the TTM trial we excluded patients who did not survive the analysis period (*n* = 62), patients with no PaCO_2_ data (*n* = 2), and patients with no data on neurological outcome (*n* = 6) (Fig. 1), leaving 869 (93%) patients for analysis. Additional data on the number of excluded patients at each measuring point are presented in Additional file 1: Table S1. Baseline characteristics of the included patients are presented in Table 1. Of 6952 analyzed measuring points, 878 (12.6%) were missing. Detailed information regarding the number of missing values at each time point is presented in Table 2 and multiple imputation was used to overcome the missingness. In total, 485 patients had no missing values. Overall, 440 (50.6%) of 869 patients had a good outcome while the outcome of 429 (49.4%) patients was considered poor.

**Table 1:** Patient baseline characteristics

Demographic characteristic	Total *n* = 869
Age (years), mean ± SD	63.9 ± 12.2
Male sex, *n* (%)	707 (81.4)
Background, *n* (%)	
Chronic heart failure	55 (6.3)
TIA or stroke	69 (8.0)
Arterial hypertension	347 (40.1)
Asthma/COPD	86 (9.9)
Diabetes mellitus	128 (14.8)
Previous PCI	101 (11.6)
Previous CABG	82 (9.5)
Cardiac arrest characteristics	
Bystander witnessed arrest, *n* (%)	783 (90.1)
Bystander CPR, *n* (%)	638 (73.4)
Shock on admission, *n* (%)	111 (12.8)
Prehospital intubation, *n* (%)	576 (67.2)
Time to ROSC (min), mean ± SD	30.4 ± 21.7
Characteristics on admission	
pH	7.21 ± 0.15
PaCO_2_ (kPa), mean ± SD	6.4 ± 2
PaO_2_ (kPa), mean ± SD	25.1 ± 17
Lactate (mmol/L), mean ± SD	6.5 ± 4.3
BE -5 or less (mmol/l), *n* (%)	579 (7.3)
GCS-Motor score 1, *n* (%)	443 (51.3)
Sedated on arrival, *n* (%)	254 (29.4)

*SD* standard deviation, *TIA* transient ischemic attack, *COPD* chronic obstructive pulmonary disease, *PCI* percutaneous coronary intervention, *CABG* coronary artery bypass graft, *CPR* cardiopulmonary resuscitation, *ROSC* return of spontaneous circulation, *PaCO*_*2*_ arterial carbon dioxide pressure, *PaO*_*2*_ arterial oxygen pressure, *kPa* kilopascal, *BE* base excess, *GCS* Glasgow Coma Scale

**Table 2:** Number of missing measurements at each time point.

	Time (h)							
	T − 1^a^	0	4	12	20	28	32	36
Missing *n*	45	154	94	91	118	117	135	124
% of total	5.18	17.7	10.8	10.4	13.6	13.5	15.5	14.2

Total *n* = 869

^a^Time at admission, after return of spontaneous circulation but before randomization

**Fig. 2:**
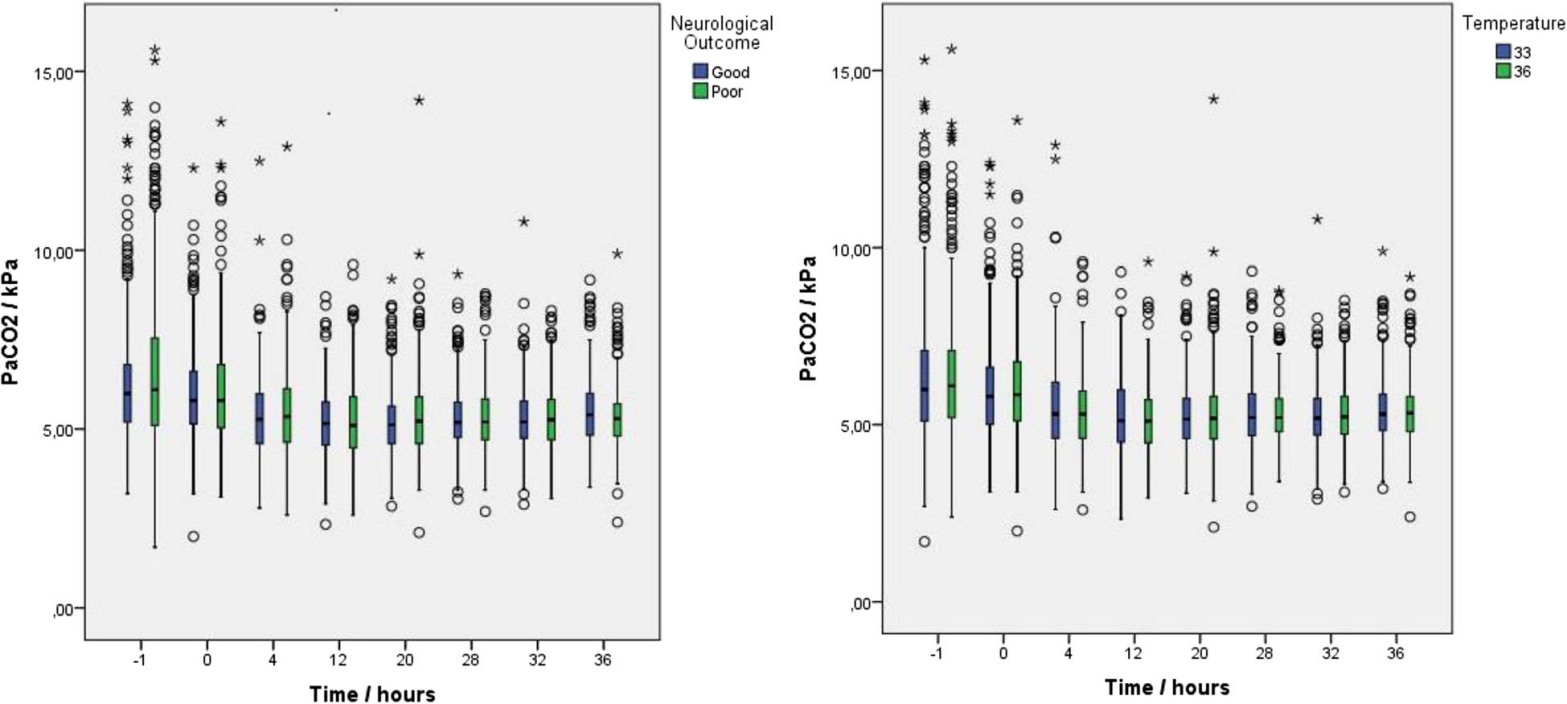
Distributional characteristics of PaCO_2_ over time. Distributional characteristics of PaCO_2_ at eight measurement points from admission to hospital to end of intervention at 36 h for TTM 33 and TTM 36 groups and investigated combined cohort dichotomized into good and poor outcome. Boxplot values displayed as median, 25% quartiles from median, and range. Core body temperature 33 °C or 36 °C. PaCO_2_ arterial carbon dioxide pressure, kPa kilopascal.

On arrival, the mean PaCO_2_ was 6.40 (SD 1.99) kPa and decreased over time in both the temperature and outcome groups (Fig. 2). In total, 516 of 869 (59%) patients were hypocapnic and 685 of 869 (79%) patients were hypercapnic at some time point during the analysis period; 371 (43%) patients were both hypocapnic and hypercapnic, and only 39 (4%) patients were normocapnic throughout. Six-month outcome data of the exposure groups are presented in Additional file 1: Table S2. The ∆PaCO_2_ group had a mean range of 2.88 (SD 1.60) kPa. The PaCO_2_ AUC for the first four measuring points was mean 5.51 (SD 0.92) kPa while the PaCO_2_ AUC for all measurements was mean 5.37 (SD 0.62) kPa.

The analysis of interaction between temperature groups (33 °C or 36 °C) and PaCO_2_ groups showed no significant difference for the main outcome (*P*_main outcome_ = 0.072–0.98) in all comparisons. The terms of interaction, except for ∆PaCO_2_ (*P* = 0.046), were also nonsignificant (*P*_interaction_ = 0.255–0.947). For the ∆PaCO_2_ group, the 33 °C and the 36 °C subgroups were analyzed separately, showing no significant association with outcome in any of the groups which made pooling of temperature groups feasible.

Multivariable models with all covariables included in the analyses for hypercapnia and hypocapnia versus normocapnia are presented in Tables 3 and 4.

**Table 3:** Multivariate model of hypercapnia versus normocapnia in relation to neurological outcome

	OR	95% CI	*P* value
Hypercapnia (normocapnia reference)	0.70	0.44–1.11	0.13
TTM group (33 °C reference)	1.00	0.71–1.42	0.99
Age (per year)	1.07	1.05–1.08	< 0.001
Sex (male reference)	1.34	0.84–2.15	0.22
Chronic heart failure (yes/no)	2.09	0.98–4.46	0.06
Asthma/COPD (yes/no)	1.32	0.72–2.43	0.37
Bystander witnessed arrest (yes/no)	0.61	0.35–1.07	0.09
Bystander CPR (yes/no)	0.87	0.57–1.33	0.53
Time to ROSC (per min)	1.03	1.02–1.04	< 0.001
GCS-Motor score (1 vs 2–5)	2.5	1.72–3.57	< 0.001
Shock on admission (yes/no)	1.56	0.88–2.75	0.13
First rhythm shockable (yes/no)	0.19	0.11–0.33	< 0.001
pH (per 1.0 unit increase)	0.28	0.07–1.17	0.08

Hypercapnia = PaCO_2_ > 6.0 kPa, normocapnia = PaCO_2_ 4.5–6.0 kPa, hypocapnia = PaCO_2_ < 4.5 kPa

OR < 1 indicates better outcome

*OR* odds ratio, *CI* confidence interval, *TTM* Target Temperature Management, *COPD* chronic obstructive pulmonary disease, *CPR* cardiopulmonary resuscitation, *ROSC* return of spontaneous circulation, *GCS* Glasgow Coma Scale

**Table 4:** Multivariate model of hypocapnia versus normocapnia in relation to neurological outcome

	OR	95% CI	*P* value
Hypocapnia (normocapnia reference)	0.96	0.64–1.45	0.85
TTM group (33 °C reference)	1.00	0.69–1.46	0.99
Age (per year)	1.06	1.04–1.08	< 0.001
Sex (male reference)	1.58	0.95–2.63	0.08
Chronic heart failure (yes/no)	1.95	0.87–4.37	0.10
Asthma/COPD (yes/no)	1.41	0.74–2.66	0.29
Bystander witnessed arrest (yes/no)	0.55	0.29–1.05	0.07
Bystander CPR (yes/no)	0.97	0.62–1.53	0.91
Time to ROSC (per min)	1.03	1.02–1.05	< 0.001
GCS-Motor score (1 vs 2–5)	1.92	1.32–2.86	0.001
Shock on admission (yes/no)	2.4	1.33–4.34	0.004
First rhythm shockable (yes/no)	0.16	0.09–0.29	< 0.001
pH (per 1.0 unit increase)	0.22	0.05–0.89	0.03

Hypercapnia = PaCO_2_ > 6.0 kPa, normocapnia = PaCO_2_ 4.5–6.0 kPa, hypocapnia = PaCO_2_ < 4.5 kPa

OR < 1 indicates better outcome

*OR* odds ratio, *CI* confidence interval, *TTM* Target Temperature Management, *COPD* chronic obstructive pulmonary disease, *CPR* cardiopulmonary resuscitation, *ROSC* return of spontaneous circulation, *GCS* Glasgow Coma Scale, *PaCO*_*2*_ arterial carbon dioxide pressure

Our main analysis revealed no statistically significant difference between the hypercapnia and normocapnia groups (OR 0.70, 95% CI 0.44–1.11; *P* = 0.13) or the hypercapnia and nonhypercapnia groups (OR 0.80, 95% CI 0.51–1.22; *P* = 0.31) in relation to poor neurological outcome. In a similar analysis, the hypocapnia group was compared to the normocapnia group and subsequently to the nonhypocapnia group with no significant differences (OR 0.96, 95% CI 0.64–1.45; *P* = 0.85 and OR 1.04, 95% CI 0.72–1.49; *P* = 0.82, respectively). The ∆PaCO_2_ analysis did not reveal a statistically significant association with poor neurological outcome, either for the 33 °C or the 36 °C subgroup (OR 1.08, 95% CI 0.9–1.29; *P* = 0.37 and OR 1.00, 95% CI 0.82–1.20; *P* = 0.96, respectively), or for the combined group (OR 1.04, 95% CI 0.91–1.18; *P* = 0.56). The PaCO_2_ AUC from admission to the end of intervention time and for the first four measured PaCO_2_ values (early dyscarbia) was not associated with poor neurological outcome (OR 1.09, 95% CI 0.83–1.42; *P* = 0.53 and OR 0.99, 95% CI 0.81–1.22; *P* = 0.96, respectively). Furthermore, we were not able to detect an association pattern with neurological outcome after we divided the total exposure time PaCO_2_ AUC into quintiles (*P* = 0.73–0.96). Details of this investigation are presented in Additional file 1: Table S5.

When analyzed separately in univariable logistic regression models, maximum PaCO_2_ as well as lowest pH showed highly significant associations with poor neurological outcome (OR 1.17, 95% CI 1.06–1.28; *P* < 0.001 and 0.03, 95% CI 0.01–0.09; *P* < 0.001, respectively). When analyzed in a combined logistic regression model, only the significant association between lowest pH and poor neurological outcome prevailed (OR 0.02, 95% CI 0.05–0.11; *P* < 0.001) per unit decrease in pH, while PaCO_2_ lost its significant association with neurological outcome (OR 0.97, 95% CI 0.86–1.09; *P* = 0.62). PaCO_2_ and pH were not highly correlated with a collinearity between the regression coefficients of − 0.64.

The TTMH analysis, comparing a normocapnia group to a mild hypercapnia group, showed a nonsignificant term of interaction (*P* = 0.79) between temperature and PaCO_2_ in relation to outcome; thus, we continued with a multivariable model without interaction. This analysis did not show a significant difference either between the mildly elevated (*n* = 121) and normocapnic (*n* = 675) PaCO_2_ groups (OR 1.01, 95% CI 0.60–1.67; *P* = 0.98) in relation to neurological outcome or between the temperature groups (OR 0.96, 95% CI 0.68–1.35; *P* = 0.83).

Of the 689 patients in the s-Tau nested cohort analysis, 100 were excluded either due to our exclusion criteria (*n* = 64) or missing peak s-Tau values (*n* = 36). The multivariable analysis of the remaining 589 patients showed no association between PaCO_2_ and s-Tau in our models (*P* = 0.12–1.00). The terms of interaction analysis were nonsignificant (*P*_interaction_ = 0.11–0.83). Complete results are presented in Table 5.

**Table 5:** Results of peak s-Tau nested cohort analysis for the employed multivariable models

Multivariable model	Estimate	95% CI	*P* value
Hypocapnia vs nonhypocapnia^a^	1.07	0.73–1.57	0.71
Hypocapnia vs normocapnia^a^	1.37	0.45–4.15	0.57
Hypercapnia vs nonhypercapnia^a^	0.68	0.42–1.10	0.12
Hypercapnia vs normocapnia^a^	1.00	0.38–2.64	1.00
Amplitude^b^	1.04	0.91–1.20	0.53
AUC, all values^b^	1.08	0.83–1.42	0.56
AUC, first four values^b^	0.94	0.76–1.17	0.59
TTMH Mild hypercapnia vs normocapnia^a^	0.75	0.43–1.28	0.29

*s-Tau* serum Tau, *CI* confidence interval, *AUC* area under curve, *TTMH* therapeutic targeted mild hypercapnia

^a^For analyses of categorical data, estimate indicates how many times higher s-Tau is compared to reference group

^b^For analyses of continuous data, estimate indicates how much higher s-Tau is per 1 kPa arterial carbon dioxide pressure increase

Both sensitivity analyses revealed similar results as the analyses on the imputed dataset, for the complete sample cohort with 485 patients (*P* = 0.32–0.96) and for the all-patient cohort with 939 patients (*P* = 0.15–0.98). For details concerning the sensitivity analyses, see Additional file 1: Tables S3 and S4.

## Discussion

In this exploratory substudy of the TTM trial, dyscarbia after ROSC was frequent. We were not able to detect a statistically significant association between hypercapnia, hypocapnia, PaCO_2_ AUC, or ∆PaCO_2_ and neurological outcome. There was no significant interaction between temperature group and carbon dioxide level in relation to outcome. PaCO_2_ was not associated with peak s-Tau levels 48 or 72 h after randomization.

Our results differ from a prospective single-center study by Roberts et al. [17] including 193 post-cardiac arrest patients, suggesting an independent association between hypocapnia and hypercapnia and poor neurological function at hospital discharge. In contrast to our study, Roberts et al. [17] included mainly patients after in-hospital cardiac arrest and used TTM in six patients only. Dyscarbia was less common compared to the present study (69% versus 96%). Our results also differ from a database study by Schneider et al. [16] analyzing PaCO_2_ values of 16,542 patients admitted after cardiac arrest that showed a higher likelihood of discharge home for the group of patients exposed to hypercapnia after ROSC compared to normocapnia or hypocapnia. As in our study, dyscarbia after ROSC was common. However, important confounders on background information on the nature of cardiac arrest (initial rhythm, time to ROSC, etc.) were not available and, apart from a nested cohort analysis, only single PaCO_2_ values were analyzed. With the exception of the CCC trial [21] randomizing to different targets of PaCO_2_, we are aware of only one study analyzing multiple PaCO_2_ values over time during the post-cardiac arrest phase [20]. This prospective observational study, including 409 OHCA patients analyzed serial blood gases during the first 24 h after ROSC and found that exposure to a moderately increased PaCO_2_ level was an independent predictor for good outcome at 12 months. We chose a comparable approach in our analysis of the PaCO_2_ AUC, but could not confirm this finding. In their study, blood gases were analyzed by either alpha-stat or pH-stat [20], while the blood gas management method employed in our study was exclusively alpha-stat. The solubility of carbon dioxide in blood is temperature dependent and might influence the ventilation strategy. Ventilation has, via the coupling of carbon dioxide and cerebral vascular tone, influence on CBF in OHCA patients treated with TTM [32]. Voicu et al. [32] showed a significant difference in PaCO_2_, arterial pH, and CBF when alpha-stat was compared to pH-stat. These findings might identify a source of error in studies using mixed blood gas management [33] and when comparing studies using different methods, which might explain the deviance of our results from other studies. pH in our study was independently associated with neurological outcome whereas maximum PaCO_2_ was not. This confirms previous findings that pH is an independent outcome predictor after ROSC [34, 35].

Our study does not indicate benefit of TTMH as investigated by Eastwood et al. [21], but also no harm. It is imperative to appreciate that we, in contrast to the pilot CCC trial [21], compared time-weighted mean PaCO_2_ values (observed, nontargeted PaCO_2_), while the CCC trial randomized patients to specific PaCO_2_ ranges (prescribed, targeted PaCO_2_). Additionally, we widened the mild hypercapnia group for our analysis to 6.0–7.30 kPa for increased robustness of our results. Whether TTMH is indeed beneficial remains to be proven in a definitive clinical trial [21]. Importantly, there was no significant interaction between temperature level and PaCO_2_ in terms of outcome, supporting the possibility to coenroll in trials investigating carbon dioxide and temperature targets [22].

The effects of PaCO_2_ on biomarkers have to date only been evaluated in the aforementioned CCC trial where mild hypercapnia reduced NSE and S100 calcium-binding protein B (S100B) levels [21]. In our cohort, PaCO_2_ showed no association with peak s-Tau levels, which is in line with the lack of association between PaCO_2_ and neurological outcome in our study. We have previously reported that s-Tau is superior to NSE in predicting outcome and that S100 does not add to a prediction model including NSE and clinical information [23, 36].

### Study strengths and limitations

There is no consensus on how to report carbon dioxide levels in relation to outcome in cardiac arrest patients and previous studies have employed methods suited to the nature of their data (single lowest/highest values versus serial measurements, within a defined time period versus not, using a prespecified sampling plan or not). In this study we have employed many different analytic approaches and used different outcomes (functional outcome and biomarkers) in order to provide an investigation as robust as possible. It is important to emphasize that the study was conceived post hoc and with a definite exploratory approach. All results must be regarded as hypothesis generating, and due to the observational design we cannot make causality statements. Blood gases represent the PaCO_2_ at a certain point in time and we assumed that the PaCO_2_ between blood samples was linear. It is also important to point out that patients not surviving the analysis period were excluded from the analysis to allow a defined exposure period of carbon dioxide. Our data were corrected for strong covariables available at admission to hospital and no covariables that may have been available later during hospital stay; therefore we did not include, for example, PaO_2_ as a covariable in our analyses. There are, however, considerable strengths in our analysis as data were derived from a large, well-controlled cohort of OHCA patients with availability of important confounders. Physiological and biochemical data were collected prospectively at specified time points according to a predefined protocol and blood gases were analyzed in a uniform way. Measurements were serial and therefore likely to demonstrate the association of PaCO_2_ with outcome in the post-cardiac arrest phase more accurately than single measurements. Follow-up data were acquired with face-to-face interviews using a structured protocol and the loss of patients in the follow-up period was minimal [24]. We performed sensitivity analyses of patients with all data registered at all sampling points and including all patients, even those not surviving the full analysis period, and obtained similar results.

## Conclusion

Dyscarbia after ROSC was common in OHCA patients, but PaCO_2_ measured as extreme values and over time was not associated with neurological outcome at 6-month follow-up. Mild hypercapnia was not associated with adverse outcome and there was no interaction with temperature group affiliation.

## Additional file

Additional file 1: Tables presenting detailed information on the excluded cohort, exposure group 6-month neurological outcome, and sensitivity analyses. (DOCX 23 kb)

## Abbreviations

AUC: Area under curve
CBF: Cerebral blood flow
CPC: Cerebral Performance Category
CPR: Cardiopulmonary resuscitation
GCS: Glasgow Coma Scale
ICU: Intensive care unit
kPa: Kilopascal
NSE: Neuron specific enolase
OHCA: Out-of-hospital cardiac arrest
OR: Odds ratio
PaCO_2_: Partial carbon dioxide pressure
PaO_2_: Arterial oxygen pressure
ROSC: Return of spontaneous circulation
S100B: S100 calcium-binding protein B
SD: Standard deviation
s-Tau: Serum Tau
TTM: Target Temperature Management
TTMH: Targeted therapeutic mild hypercapnia

## References

[1] Berdowski J, Berg RA, Tijssen JG, Koster RW (2010). Global incidences of out-of-hospital cardiac arrest and survival rates: systematic review of 67 prospective studies. Resuscitation.

[2] Mozaffarian D, Benjamin EJ, Go AS, Arnett DK, Blaha MJ, Cushman M, Das SR, de Ferranti S, Despres JP, Fullerton HJ (2016). Heart disease and stroke statistics—2016 update: a report from the American Heart Association. Circulation.

[3] Kudenchuk PJ, Sandroni C, Drinhaus HR, Bottiger BW, Cariou A, Sunde K, Dworschak M, Taccone FS, Deye N, Friberg H (2015). Breakthrough in cardiac arrest: reports from the 4th Paris International Conference. Ann Intensive Care.

[4] Kragholm K, Wissenberg M, Mortensen RN, Hansen SM, Malta Hansen C, Thorsteinsson K, Rajan S, Lippert F, Folke F, Gislason G (2017). Bystander efforts and 1-year outcomes in out-of-hospital cardiac arrest. N Engl J Med.

[5] Adielsson A, Hollenberg J, Karlsson T, Lindqvist J, Lundin S, Silfverstolpe J, Svensson L, Herlitz J (2011). Increase in survival and bystander CPR in out-of-hospital shockable arrhythmia: bystander CPR and female gender are predictors of improved outcome. Experiences from Sweden in an 18-year perspective. Heart.

[6] Neumar RW, Eigel B, Callaway CW, Estes NA, Jollis JG, Kleinman ME, Morrison LJ, Peberdy MA, Rabinstein A, Rea TD (2015). American Heart Association response to the 2015 Institute of Medicine Report on strategies to improve cardiac arrest survival. Circulation.

[7] van Diepen S, Girotra S, Abella BS, Becker LB, Bobrow BJ, Chan PS, Fahrenbruch C, Granger CB, Jollis JG, McNally B (2017). Multistate 5-year initiative to improve Care for out-of-Hospital Cardiac Arrest: primary results from the HeartRescue project. J Am Heart Assoc.

[8] Reynolds JC, Lawner BJ (2012). Management of the post-cardiac arrest syndrome. J Emerg Med.

[9] Nolan JP, Soar J, Zideman DA, Biarent D, Bossaert LL, Deakin C, Koster RW, Wyllie J, Bottiger B (2010). European Resuscitation Council Guidelines for Resuscitation 2010 Section 1. Executive summary. Resuscitation.

[10] Hazinski MF, Nolan JP, Billi JE, Bottiger BW, Bossaert L, de Caen AR, Deakin CD, Drajer S, Eigel B, Hickey RW (2010). Part 1: executive summary: 2010 international consensus on cardiopulmonary resuscitation and emergency cardiovascular care science with treatment recommendations. Circulation.

[11] Aufderheide TP, Lurie KG (2004). Death by hyperventilation: a common and life-threatening problem during cardiopulmonary resuscitation. Crit Care Med.

[12] Falkenbach P, Kamarainen A, Makela A, Kurola J, Varpula T, Ala-Kokko T, Perttila J, Tenhunen J (2009). Incidence of iatrogenic dyscarbia during mild therapeutic hypothermia after successful resuscitation from out-of-hospital cardiac arrest. Resuscitation.

[13] Kety SS, Schmidt CF (1948). The effects of altered arterial tensions of carbon dioxide and oxygen on cerebral blood flow and cerebral oxygen consumption of normal young men. J Clin Invest.

[14] Brackett NC, Cohen JJ, Schwartz WB (1965). Carbon dioxide titration curve of normal man. Effect of increasing degrees of acute hypercapnia on acid-base equilibrium. N Engl J Med.

[15] Laffey JG, Kavanagh BP (2002). Hypocapnia. N Engl J Med.

[16] Schneider AG, Eastwood GM, Bellomo R, Bailey M, Lipcsey M, Pilcher D, Young P, Stow P, Santamaria J, Stachowski E (2013). Arterial carbon dioxide tension and outcome in patients admitted to the intensive care unit after cardiac arrest. Resuscitation.

[17] Roberts BW, Kilgannon JH, Chansky ME, Mittal N, Wooden J, Trzeciak S (2013). Association between postresuscitation partial pressure of arterial carbon dioxide and neurological outcome in patients with post-cardiac arrest syndrome. Circulation.

[18] Roberts BW, Kilgannon JH, Chansky ME, Trzeciak S (2014). Association between initial prescribed minute ventilation and post-resuscitation partial pressure of arterial carbon dioxide in patients with post-cardiac arrest syndrome. Ann Intensive Care.

[19] Vannucci RC, Towfighi J, Heitjan DF, Brucklacher RM (1995). Carbon dioxide protects the perinatal brain from hypoxic-ischemic damage: an experimental study in the immature rat. Pediatrics.

[20] Vaahersalo J, Bendel S, Reinikainen M, Kurola J, Tiainen M, Raj R, Pettila V, Varpula T, Skrifvars MB, FINNRESUSCI Study Group. Arterial blood gas tensions after resuscitation from out-of-hospital cardiac arrest: associations with long-term neurological outcome. Crit Care Med. 2014;42(6):1463–70. 10.1097/CCM.0000000000000228 24557423

[21] Eastwood GM, Schneider AG, Suzuki S, Peck L, Young H, Tanaka A, Martensson J, Warrillow S, McGuinness S, Parke R (2016). Targeted therapeutic mild hypercapnia after cardiac arrest: a phase II multi-centre randomised controlled trial (the CCC trial). Resuscitation.

[22] Parke RL, McGuinness S, Eastwood GM, Nichol A, Nielsen N, Dankiewicz J, Bellomo R (2017). Co-enrolment for the TAME and TTM-2 trials: the cerebral option. Crit Care Resusc.

[23] Mattsson N, Zetterberg H, Nielsen N, Blennow K, Dankiewicz J, Friberg H, Lilja G, Insel PS, Rylander C, Stammet P (2017). Serum tau and neurological outcome in cardiac arrest. Ann Neurol.

[24] Nielsen N, Wetterslev J, Cronberg T, Erlinge D, Gasche Y, Hassager C, Horn J, Hovdenes J, Kjaergaard J, Kuiper M, et al. Targeted temperature management at 33 degrees C versus 36 degrees C after cardiac arrest. N Engl J Med. 2013;369(23):2197–206. 10.1056/NEJMoa1310519 24237006

[25] Jacobs I, Nadkarni V, Bahr J, Berg RA, Billi JE, Bossaert L, Cassan P, Coovadia A, D'Este K, Finn J (2004). Cardiac arrest and cardiopulmonary resuscitation outcome reports: update and simplification of the Utstein templates for resuscitation registries: a statement for healthcare professionals from a task force of the international liaison committee on resuscitation (American Heart Association, European Resuscitation Council, Australian Resuscitation Council, New Zealand Resuscitation Council, Heart and Stroke Foundation of Canada, InterAmerican Heart Foundation, Resuscitation Councils of Southern Africa). Circulation.

[26] Brain Resuscitation Clinical Trial I Study Group (1986). Randomized clinical study of thiopental loading in comatose survivors of cardiac arrest. N Engl J Med.

[27] Jennett B, Bond M (1975). Assessment of outcome after severe brain damage. Lancet.

[28] Phelps R, Dumas F, Maynard C, Silver J, Rea T (2013). Cerebral performance category and long-term prognosis following out-of-hospital cardiac arrest. Crit Care Med.

[29] Blondin NA, Greer DM (2011). Neurologic prognosis in cardiac arrest patients treated with therapeutic hypothermia. Neurologist.

[30] Marshall A, Altman DG, Holder RL, Royston P (2009). Combining estimates of interest in prognostic modelling studies after multiple imputation: current practice and guidelines. BMC Med Res Methodol.

[31] van Buuren S, Groothuis-Oudshoorn K. mice: Multivariate Imputation by Chained Equations in R. J stat soft. 2011;45(3):67.

[32] Voicu S, Deye N, Malissin I, Vigue B, Brun PY, Haik W, Champion S, Megarbane B, Sideris G, Mebazaa A (2014). Influence of alpha-stat and pH-stat blood gas management strategies on cerebral blood flow and oxygenation in patients treated with therapeutic hypothermia after out-of-hospital cardiac arrest: a crossover study. Crit Care Med.

[33] Eastwood GM, Suzuki S, Lluch C, Schneider AG, Bellomo R (2015). A pilot assessment of alpha-stat vs pH-stat arterial blood gas analysis after cardiac arrest. J Crit Care.

[34] Momiyama Y, Yamada W, Miyata K, Miura K, Fukuda T, Fuse J, Kikuno T (2017). Prognostic values of blood pH and lactate levels in patients resuscitated from out-of-hospital cardiac arrest. Acute Med Surg.

[35] Maupain C, Bougouin W, Lamhaut L, Deye N, Diehl JL, Geri G, Perier MC, Beganton F, Marijon E, Jouven X (2016). The CAHP (Cardiac Arrest Hospital Prognosis) score: a tool for risk stratification after out-of-hospital cardiac arrest. Eur Heart J.

[36] Stammet P, Dankiewicz J, Nielsen N, Fays F, Collignon O, Hassager C, Wanscher M, Unden J, Wetterslev J, Pellis T (2017). Protein S100 as outcome predictor after out-of-hospital cardiac arrest and targeted temperature management at 33 degrees C and 36 degrees C. Crit Care.

