# Supplementary material for: Carbon dioxide dynamics in relation to neurological outcome in resuscitated out-of-hospital cardiac arrest patients: an exploratory Target Temperature Management Trial substudy
Source: Crit Care. 2018 Aug 18;22:196. doi: 10.1186/s13054-018-2119-5 (PMC6098627; doi:10.1186/s13054-018-2119-5)
Supplement: Supplementary file 1 — Tables presenting detailed information on the excluded cohort, exposure group 6-month neurological outcome, and sensitivity analyses. (DOCX 23 kb) [file 13054_2018_2119_MOESM1_ESM.docx]

**Supplementary information**

**Carbon dioxide dynamics in relation to neurological outcome in resuscitated out-of-hospital cardiac arrest patients – an exploratory Target Temperature Management Trial substudy**

| **Table S1. Number of patients excluded and PaCO_2_ at each measuring point.** | | | | | | | | |
| --- | --- | --- | --- | --- | --- | --- | --- | --- |
| Time | T -1 | T 0 | T 4 | T 12 | T 20 | T 28 | T 32 | T 36 |
| N Valid | 59 | 50 | 38 | 26 | 18 | 7 | 3 | 2 |
| N Missing | 11 | 20 | 32 | 44 | 52 | 63 | 67 | 68 |
| PaCO_2_ mean (kPa) | 8.07 | 6.38 | 6.12 | 5.37 | 5.96 | 6.21 | 5.44 | 5.65 |
| PaCO_2_ median (kPa) | 7.10 | 6.13 | 5.62 | 5.04 | 5.35 | 5.80 | 5.70 | 5.65 |
| Standard deviation | 3.69 | 1.79 | 1.87 | 1.51 | 2.19 | 2.61 | 1.10 | 0.49 |
| Minimum (kPa) | 3.30 | 3.50 | 3.40 | 3.06 | 3.00 | 4.18 | 4.23 | 5.30 |
| Maximum (kPa) | 20.00 | 12.90 | 11.20 | 10.00 | 11.40 | 11.90 | 6.38 | 6.00 |
| Percentile 25 (kPa) | 5.80 | 5.37 | 4.60 | 4.20 | 4.40 | 4.60 | 4.23 | 5.30 |
| Percentile 50 (kPa) | 7.10 | 6.14 | 5.62 | 5.04 | 5.35 | 5.80 | 5.70 | 5.65 |
| Percentile 75 (kPa) | 9.40 | 7.23 | 7.40 | 6.21 | 6.95 | 6.10 |  |  |

*N = Number. T = measuring point in hours before/after randomization. PaCO_2_ = partial arterial carbon dioxide pressure. kPa = kilopascal*

| **Table S2. 6-month neurological outcome in PaCO_2_ extreme value exposure groups dichotomized to good and poor. n=869** | | | |
| --- | --- | --- | --- |
| PaCO_2_ Group | Total n (%) | Good outcome n (%) | Poor outcome n (%) |
| Hypercapnia (> 6.0 kPa) | 685 (79) | 349 (51) | 336 (49) |
| Hypocapnia (< 4.5 kPa) | 516 (59) | 260 (50) | 256 (50) |
| Normocapnia (4.5 – 6.0 kPa) | 39 (4) | 23 (59) | 16 (41) |
| Hyper- and hypocapnia | 371 (43) | 192 (52) | 179 (48) |

*n = number. PaCO_2_ = partial arterial carbon dioxide pressure. kPa = kilopascal. CPC = Cerebral performance category. Good outcome = CPC 1 and 2, poor outcome = CPC 3-5. CPC 1 good cerebral performance, CPC 2 moderate cerebral disability, independent in activities of daily life, CPC 3 severe cerebral disability, dependent on others for daily support, 4 vegetative state and CPC 5 dead.*

| **Table S3. Sensitivity analysis of PaCO_2_ groups including all patients (n=939), adjusted for confounders.** | | | |
| --- | --- | --- | --- |
| Analysis | **OR** | **95% CI** | **p-value** |
| Hypercapnia vs normocapnia | 0.70 | 0.30 - 1.64 | 0.41 |
| Hypercapnia vs non-hypercapnia | 0.73 | 0.48 - 1.12 | 0.15 |
| Hypocapnia vs normocapnia | 1.04 | 0.41 - 2.60 | 0.94 |
| Hypocapnia vs non-hypocapnia | 0.99 | 0.70 - 1.41 | 0.96 |
| PaCO_2_-AUC first four measurements | 1.03 | 0.86 - 1.24 | 0.71 |
| PaCO_2_-AUC all measurements | 1.09 | 0.89 - 1.34 | 0.40 |
| Amplitude | 1.00 | 0.88 - 1.13 | 0.98 |

*n = number. OR = Odds Ratio. CI = Confidence Interval. PaCO_2_ = partial arterial carbon dioxide pressure. AUC = Area under curve. OR < 1 indicates better outcome. Patients with no outcome data (n=6), no PaCO_2_ data (n=2) and lack of confounders (n=5) were excluded, 926 patients we included in the final analysis. Confounders corrected for: age (years), sex (male/female), chronic heart failure (yes/no), asthma/chronic obstructive pulmonary disease (yes/no), cardiac arrest witnessed (yes/no), bystander CPR (yes/no), first rhythm shockable (yes/no), time to ROSC (minutes), GCS-Motor Score (1 versus 2 - 5), shock on admission (yes/no), pH at admission (units).*

| **Table S4. Sensitivity analysis of PaCO_2_ groups including complete cases (n=485), adjusted for confounders.** | | | |
| --- | --- | --- | --- |
| Analysis | **OR** | **95% CI** | **p-value** |
| Hypercapnia vs normocapnia | 0.60 | 0.21 – 1.68 | 0.33 |
| Hypercapnia vs non-hypercapnia | 0.76 | 0.44 – 1.30 | 0.32 |
| Hypocapnia vs normocapnia | 1.21 | 0.41 – 3.56 | 0.73 |
| Hypocapnia vs non-hypocapnia | 1.19 | 0.76 – 1.87 | 0.45 |
| PaCO_2_-AUC first four measurements | 0.97 | 0.74 – 1.27 | 0.84 |
| PaCO_2_-AUC all measurements | 1.00 | 0.71 – 1.43 | 0.96 |
| Amplitude | 1.06 | 0.89 – 1.24 | 0.52 |

*n = number. OR = Odds Ratio. CI = Confidence Interval. PaCO_2_ = partial arterial carbon dioxide pressure. AUC = Area under curve. OR < 1 indicates better outcome. Confounders corrected for: age (years), sex (male/female), chronic heart failure (yes/no), asthma/chronic obstructive pulmonary disease (yes/no), cardiac arrest witnessed (yes/no), bystander CPR (yes/no), first rhythm shockable (yes/no), time to ROSC (minutes), GCS-Motor Score (1 versus 2 - 5), shock on admission (yes/no), pH at admission (units).*

| **Table S5. Outcome of the time weighted mean PaCO_2_ values divided into quintiles with outcome in quintile 1 as reference.** | | | | |
| --- | --- | --- | --- | --- |
|  | **PaCO_2_ (kPa)** | **OR** | **95% CI** | **p-value** |
| Quintile 1 | <4.84 | reference |  |  |
| Quintile 2 | 4.84 – 5.15 | 0.90 | 0.51 – 1.60 | 0.731 |
| Quintile 3 | 5.15 – 5.44 | 0.96 | 0.55 – 1.70 | 0.900 |
| Quintile 4 | 5.44 – 5.86 | 1.03 | 0.59 – 1.80 | 0.920 |
| Quintile 5 | >5.86 | 1.01 | 0.57 – 1.79 | 0.964 |

*OR = Odds Ratio. CI = Confidence Interval. PaCO_2_ = partial arterial carbon dioxide pressure. OR < 1 indicates better outcome. Data was corrected for confounders age (years), sex (male/female), chronic heart failure (yes/no), asthma/chronic obstructive pulmonary disease (yes/no), cardiac arrest witnessed (yes/no), bystander CPR (yes/no), first rhythm shockable (yes/no), time to ROSC (minutes), GCS-Motor Score (1 versus 2 - 5), shock on admission (yes/no), pH at admission (units).*
